# The future burden of cancer in England: incidence and numbers of new patients in 2020

**DOI:** 10.1038/sj.bjc.6603746

**Published:** 2007-05-01

**Authors:** H Møller, L Fairley, V Coupland, C Okello, M Green, D Forman, B Møller, F Bray

**Affiliations:** 1 King's College London, Thames Cancer Registry, 42 Weston Street, London SE1 3QD, UK; 2 Northern and Yorkshire Cancer Registry and Information Service, Arthington House, Cookridge Hospital, Leeds LS16 6QB, UK; 3 Standards and Quality Analytical Team, Department of Health, Area 403, Wellington House, 135-155 Waterloo Road, London SE1 8UG, UK; 4 Cancer Registry of Norway, Montebello, N-0310 Oslo, Norway

**Keywords:** epidemiology, projections, burden of disease, England

## Abstract

We estimated the future cancer incidence rates and the future numbers of cancer cases in England up to 2020 using cancer registration data for 1974–2003, and the official population projections from ONS up to 2023. Data were analysed using an age-period-cohort model as developed for the Nordic countries. We predict that for all cancers combined there will be relatively little change in age-standardised incidence rates in 2020. The number of new cancer cases per year in England is, however, predicted to increase by 33%, from 224 000 in 2001 to 299 000 cases in 2020. This increase is mainly due to the anticipated effects of population growth and ageing; cancer patients in 2020 will be older than today's cancer population.

We can quantify the future burden of cancer from two different perspectives. Firstly, age-standardised rates describe the occurrence of cancer on a *per capita* basis, taking account of changes in age composition and size of the population (Parkin, 2006). Secondly, from the point of view of cancer care and cancer services provision, the burden of cancer is more usefully measured as the total number of persons with cancer who require diagnostic, therapeutic, supportive or palliative services (Bray and Møller, 2006).

Several large studies have forecast future cancer rates and numbers, using a variety of statistical methods (Møller *et al*, 2002; New Zealand Ministry of Health, 2002; Scottish Executive, 2004; Clements *et al*, 2005). The most common methods relate incidence to the three interdependent time dimensions of age, calendar period and generation. When cancer rates have changed linearly in recent periods and in consecutive generations, it is reasonable to expect the change to continue, at least to some degree and for some time. When particularly low or high rates are observed in a recent generation at a young age, it may be reasonable to assume that this generation will be subject to similar rates when they become older. Future changes are only predictable if they are linear extensions of past trends; otherwise, future period effects are not predictable from past rates. This is a major source of uncertainty in cancer forecasts and is the main reason why many predictions turn out to be too low or too high.

## SUBJECTS AND METHODS

For this analysis of the future cancer rates and numbers of cancer cases in England, we have used a method of estimation that was developed in a comprehensive and systematic analysis of cancer trends in the Nordic countries (Møller *et al*, 2002, 2003). Møller and co-workers used the long data series in the Nordic countries to develop a large number of predictions of present rates as would have been forecast 20 years ago, and compared the predicted rates with those actually observed, identifying a set of analysis options that tended to give the most accurate predictions. Our analysis of the English data adopts the Nordic method of estimation and the standard set of recommendations with very few modifications. The analysis makes no assumption about changes in exposure to risk factors, but relies entirely on the extrapolation of the recorded rates in the past.

Data on cancer incidence counts and corresponding population denominators in England were obtained from the Office for National Statistics. The data were aggregated into 5-year periods (1974–1978, … 1999–2003) and 5-year age groups (0–4, 5–9, … 80–84, 85+) by sex. Cancers were categorised into 21 commonly used types on the basis of ICD9 and ICD10 codes; remaining types were categorised as ‘other sites’ and analysed as a separate group when summing up to the figures for all cancers combined. Population figures consisted of forecasts for four future 5-year periods (2004–2008, 2009–2013, 2014–2018, 2019–2023) in 5-year age groups by sex. As an approximation, these 5-year periods were used to represent the single years 2005, 2010, 2015 and 2020, respectively. The predicted cancer burden was measured by the numbers of cancer cases in future calendar periods and calculated by first projecting the observed cancer incidence trends, then multiplying these predicted incidence rates by the forecast populations in these periods.

Incidence rates were modelled as a function of age, calendar period and birth cohort, with the central value of the latter calculated by subtracting the midpoint of each age category from the midpoint of the period. A recent approach to projecting incidence rates (Møller *et al*, 2002) was adapted for most of the cancer sites, based on a standard age-period-cohort model (Osmond, 1985), but with some modifications which have been shown empirically to improve the predictions (Møller *et al*, 2003). The multiplicative relationship between incidence and the covariates in the standard model produces predictions in which the rates change exponentially with time. The first modification was to use a power link function instead of the default logarithmic link function in Poisson regression as this has been demonstrated to level off the exponential growth rates. The model can be written as *R_ap_* = (*A_a_* + *D* · *p* + *P_p_* + *C_c_*)^5^, where *R*_*ap*_ is the incidence rate in age group *a* in calendar period *p*, *A*_*a*_ is the age component for age group *a*, *D* is the common drift parameter which summarises the linear component of the trend, which cannot be attributed to either period or cohort (Clayton and Schifflers, 1987). *P*_*p*_ is the nonlinear period component of period *p* and *C*_*c*_ is the nonlinear cohort component of cohort *c*. The linear component *D* and the nonlinear cohort effects were projected. To allow for a damping of the impact of current trends in the future time periods, a gradual reduction in the drift parameter of 25, 50 and 75% in the second, third and fourth 5-year period, respectively, was used. Misleading predictions can occur if there is a recent sharp change in the trends, and the average increase over the entire observation period is projected. To improve accuracy, the trend in the most recent 10 years was used as the drift component to be projected in situations where the rates had statistically significant curvature in the prediction base.

For cancers of the prostate and bladder, future rates were not based on extrapolation of past trends, but by assuming that the rates would remain unchanged from those in the most recent period, 1999–2003. Extrapolation of trends could not be justified, as recent artefactual changes were considered unlikely to continue (see Discussion).

We summarised the results using the cumulative risk to age 75 years, the age-standardised incidence rates using the European standard population (Day, 1987), and the future numbers of new cancer cases (in thousands per year). We considered the changes in these parameters, both in absolute and in relative terms. The percentage change in the annual number of cases from 2001 to 2020 was divided into one part due to increased risk of being diagnosed with cancer, and another due to changes in the population size and age distribution (Møller *et al*, 2002). The prediction package NORDPRED written in R is available online (Nordpred, 2006).

## RESULTS

**Figure 1:**
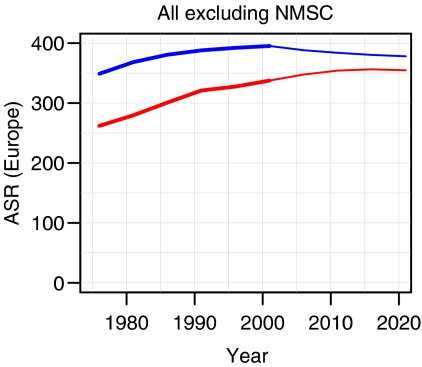
Trends in age-standardised (European standard population) incidence rates of all cancers combined (excluding non-melanoma skin cancer) up to 2020. Rates in males in blue; rates in females in red.

**Figure 2:**
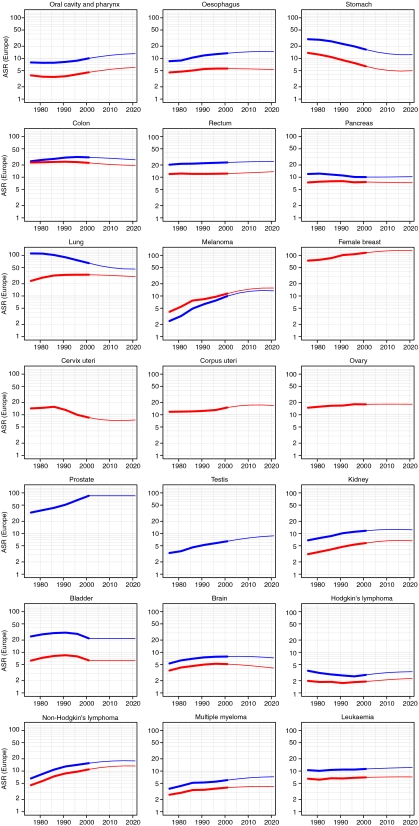
Trends in age-standardised (European standard population) incidence rates of different cancers and predictions up to 2020. Rates in males in blue; rates in females in red.

Considered overall, the age-standardised incidence rates of all cancers combined (excluding non-melanoma skin cancer) are predicted to decrease in males and increase slightly in females (Figure 1). Figure 2 shows the age-standardised incidence rates for different cancers. The blue lines denote the rates in men and the red lines the rates in women. The empirical rates up to 2003 are shown with a thicker line than the predicted rates from 2004 to 2021. The *Y*-axis is logarithmic and identical in all the individual diagrams. The analysis predicts increasing rates in several cancers: oral cavity and pharynx, melanoma, testis (all with increases of 30–40%) and non-Hodgkin's lymphoma (10–20% increase). Decreasing rates are predicted for cancers of the stomach, colon, lung, cervix and brain (decreases of 10–25%).

Table 1 shows the forecast change in numbers of cancer cases from 2001 to 2020. In contrast to the predicted future incidence rates, the numbers of cancer cases in the English population are predicted to increase very substantially from around 224 000 in 2001 to around 299 000 in 2020. This is an increase of 33% (36% in males; 30% in females). The main reason is the increasing population, especially of middle-aged and old people.

Table 1 also shows the change in numbers of different cancers and breaks down the increases into the contribution from change in incidence and change in the population at risk. For most cancers, the effect of demography is stronger than the effect of changing incidence rates. Notable exceptions are oral and pharyngeal cancer, melanoma, testis cancer and Hodgkin's lymphoma where the changes in incidence are greater than the change in population. For cancers of the breast, uterine corpus and kidney (in females), the contributions of incidence rate and population are of similar magnitude.

## DISCUSSION

Among systematic studies of national and international cancer incidence trends (Hakulinen *et al*, 1986; Coleman *et al*, 1993; Quinn *et al*, 2001; Scottish Executive, 2004), the general pattern in past decades has been an increase in the age-standardised incidence rates of all cancers combined. With respect to cancer prevention, our study has shown that a turning point may have been reached or is soon to be reached, and that cancer rates in England are now decreasing in males and are predicted to start decreasing from 2015 in females. The single most important factor in this stabilisation is probably the reduction in smoking prevalence. Cancer will continue to affect the lives of a great many people in England, emphasising the continued need for primary prevention. Although preventive interventions may have a material impact on particular types of cancer with known, strong and preventable causes (such as lung cancer, oral cancer and cervix cancer), the general impact of primary prevention on the total age-adjusted cancer incidence in the next decades will probably be relatively small unless new and powerful preventive measures are discovered and put into practice.

The predicted growth in the total annual number of cancer cases in the population represents an important gradual increase in the workload and resource requirements for cancer care services in the coming years. Much of the predicted growth will take place in the older age groups, for which cancer services will need to be developed (Haward, 2006). Future cancer services provision should give consideration to the special circumstances of this group, including increased surgical mortality, toxicity of chemotherapy, treatment-related complications, general comorbidity, and special needs for supportive and palliative care (Edwards *et al*, 2002; Balducci and Ershler, 2005). In parallel with the increasing number of cancer patients, it is likely that patients' expectations, professional consensus about best practice, official guidelines and innovations in diagnostics and therapy will lead to significantly increasing intensity and costs of care for the individual patient and for society as a whole.

We should emphasise that the predicted rates and numbers of cases are uncertain and depend on an assumption about the continuity of past trends. In the analysis of the earlier predictions in the Nordic countries (Møller *et al*, 2003), the errors of predicted numbers compared with actual numbers observed subsequently were typically 10–20% on either side. A similar analysis in Scotland showed that half of the predicted future numbers were incorrect by more than 10% and a fifth by more than 20% (Scottish Executive, 2004). We have compared the present results with corresponding estimates from a simpler method of linear extrapolation of age-specific rates that was made available by Dyba and Hakulinen (ENCR, 2006). Our method gave marginally higher estimates of future numbers of cancer cases, probably because it relies more on the cohort component (current rates in the youngest age groups) and less on the linear extrapolation of rates in all age groups (data not shown).

We think these predictions are the most plausible at present, but those for individual sites will probably deviate from the actual rates in the 2020s, to the order of 10–20% to either side, and for a few cancers it will be larger than that. For all cancers combined, the error should be lower because too high and too low predictions for particular sites will tend to cancel out, but margins of error of 5% in the rates and 10% in the numbers of cases would seem likely.

Population forecasting is required for accurate estimates of the future events from projected rates. However, forecasted population data are, by their nature, predictions themselves, based on forecasted birth and death rates and immigration and emigration levels.

Our estimates are intentionally presented without standard errors or confidence intervals, as these would be extremely low because of the large size of the population. The uncertainty associated with these predictions does not concern sampling error but the unquantifiable bias when trends in some cancers behave in a manner that is inconsistent with the assumptions of the statistical analysis of past rates.

The single most critical aspect of these predictions concerns the future rates and numbers of prostate cancer. The standard set of assumptions would predict a doubling of the age-standardised incidence of prostate cancer, which we consider unlikely, and we decided simply to assume that the age-specific rates in 1999–2003 would remain constant, so that future numbers of prostate cancer would increase only in consequence of demographic changes in the population. Recent trends in prostate cancer in England are complex, with increases in the relatively young age groups (up to 69 years) due principally to increasing use of the prostate-specific antigen (PSA) test. However, rates have so far declined or remained constant in the older age groups because of increasing medical treatment for benign urinary obstruction and resulting decrease in use of transurethral resection, which had previously led to many prostate cancer diagnoses (Evans and Møller, 2003): the future use of the PSA test will be critical. If it stabilises at the current level, our estimates will be correct or even a little high, but if PSA testing in asymptomatic older men increases, the prostate cancer rates and numbers may increase above our predictions.

To illustrate the sensitivity of our predictions, we assumed that age-specific rates increased by 50% in all age groups, resulting in the age-standardised rate of all male cancers combined increasing by 6% (not 4%), future numbers of prostate cancer by 123% (not 48%) and future numbers of all male cancers combined by 53% (not 36%).

We assumed that future bladder cancer rates would remain constant. With changes in classification of bladder neoplasms, a marked recent decrease in bladder cancer incidence is evident (Figure 1). Previously, many *in situ* lesions were counted as invasive bladder cancer. Available data did not permit a proper reclassification to a uniform concept of bladder cancer. Rather than allowing this artefact to predict a strong decrease, we decided to compel the rates to remain constant. The implications of this assumption about bladder cancer are less marked than was the case for prostate cancer.

In summary, the present study highlights the need for early reassessment of resources and infrastructure for cancer control and care, in order to anticipate the increasing number of cancer patients.

## Figures and Tables

**Table 1:** Recorded cancer incidence in males and females in England 2001, predicted cancer incidence around 2020 and the corresponding percentage change in incidence, decomposed into changing risk and demographic components

		**Males**					**Females**				
**ICD-10 code**	**Cancer type**	**Number of cancer cases in 2001^a^**	**Number of cancer cases in 2020^b^**	**Change overall (%)^c^**	**Change due to change in risk (%)^d^**	**Change due to change in population (%)^e^**	**Number of cancer cases in 2001^a^**	**Number of cancer cases in 2020^b^**	**Change overall (%)^c^**	**Change due to change in risk (%)^d^**	**Change due to change in population (%)^e^**
C00-C14	Lip, mouth, pharynx	2624	4584	75	43	32	1458	2290	57	34	23
C15	Oesophagus	3771	5974	58	15	43	2293	2770	21	−6	26
C16	Stomach	4780	5046	6	−42	47	2649	2468	−7	−33	26
C18	Colon	8872	11 692	32	−15	46	8746	9786	12	−14	26
C19–C21	Rectum	6468	9842	52	10	42	4503	6090	35	10	25
C25	Pancreas	2852	4198	47	2	45	3022	3683	22	−4	26
C33–C34	Lung	18 495	18 519	0	−45	45	12 004	13 600	13	−12	26
C43	Melanoma	2629	4942	88	58	30	3377	5608	66	49	17
C50	Breast	—	—	—	—	—	34 636	49 743	44	22	21
C53	Cervix uteri	—	—	—	—	—	2420	2123	−12	−24	12
C54	Corpus uteri	—	—	—	—	—	4684	7149	53	27	25
C56–C57	Ovary	—	—	—	—	—	5612	6933	24	1	23
C61	Prostate^f^	24 717	36 703	48	0	48	—	—	—	—	—
C62	Testis	1600	2332	46	39	7	—	—	—	—	—
C64–C66	Kidney	3199	4790	50	12	38	1967	2955	50	26	24
C67	Bladder^f^	6394	9547	49	0	49	2582	3266	26	0	26
C71	Brain	2033	2414	19	−8	26	1501	1448	−4	−23	19
C81	Hodgkin's lymphoma	690	939	36	22	14	497	636	28	18	10
C82–C85	NHL	4237	6748	59	24	36	3681	5757	56	33	23
C88, C90	Myeloma	1701	2954	74	28	46	1480	2006	36	10	26
C91–C95	Leukaemia	3084	4687	52	12	40	2409	3080	28	5	22
	Other sites	13 493	16 470	22	−21	43	12 956	15 107	17	−8	24
C00–C97 (excluding C44)	All excluding NMSC^g^	111 639	152 381	36	−7	43	112 477	146 500	30	7	23

NHL=non-Hodgkin's lymphoma; NMSC=non-melanoma skin cancer.

a Average annual incidence as recorded 1999–2003.

b Average annual incidence as predicted 2019–2023.

c % change in the number of new cases predicted for 2019–2023 compared to 1999–2003.

d % change in the number of new cases due to changes in risk.

e % change in the number of new cases due to changes in population age structure and size.

f Incidence rates were assumed to remain constant for prostate and bladder cancer.

g All cancers excluding non-melanoma cancers of skin; numbers and proportional changes based on combining the specific cancer sites.
